# Supplementary material for: The causal role of circulating inflammatory markers in osteoporosis: a bidirectional Mendelian randomized study
Source: Front Immunol. 2024 Jul 18;15:1412298. doi: 10.3389/fimmu.2024.1412298 (PMC11291241; doi:10.3389/fimmu.2024.1412298)
Supplement: Supplementary file 1 [file DataSheet_1.zip › supplementary material/CIM-OSTEOPOROSIS /GCST90274828.csv_leave_one_out.pdf]

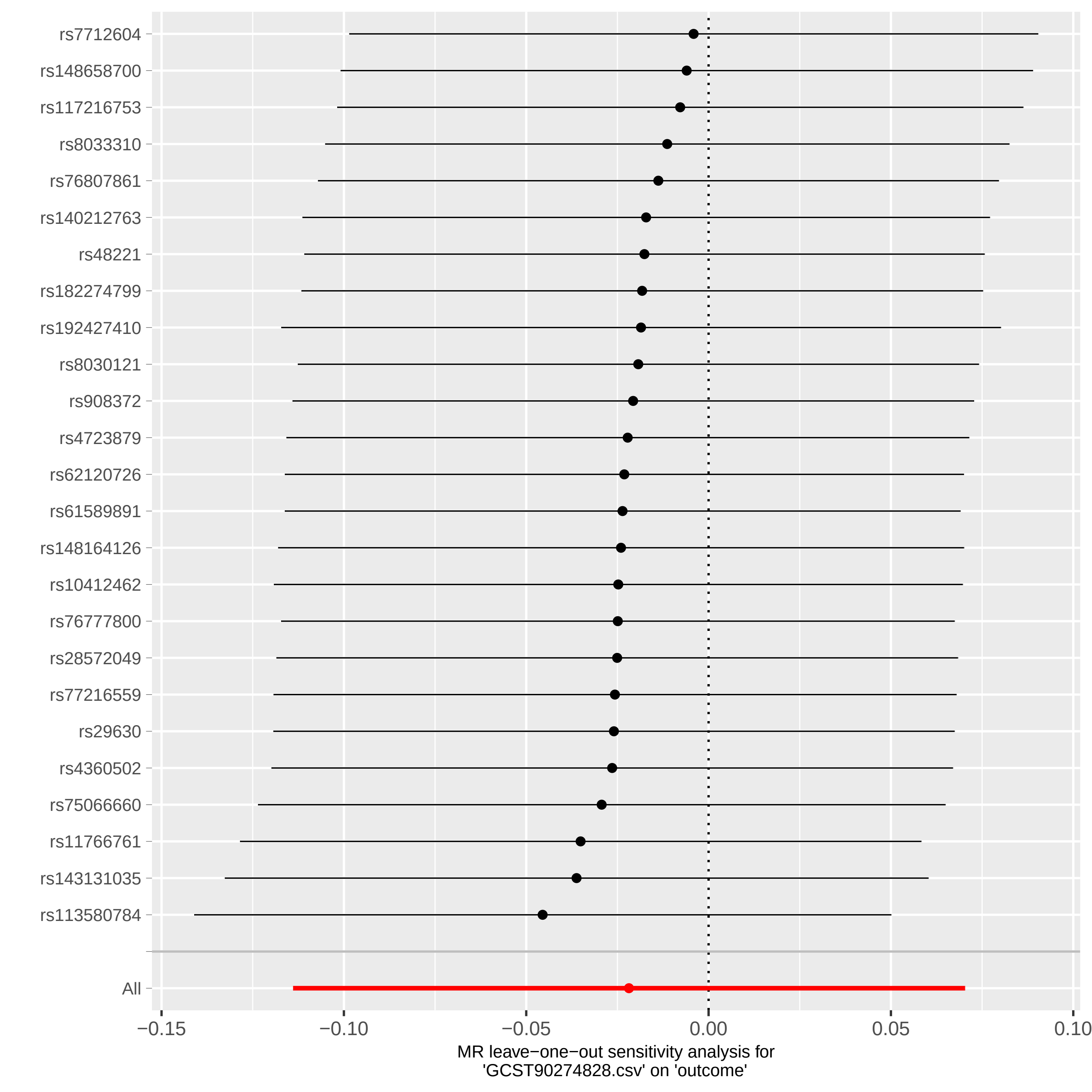
rs7712604
rs148658700
rs117216753
rs8033310
rs76807861
rs140212763
rs48221
rs182274799
rs192427410
rs8030121
rs908372
rs4723879
rs62120726
rs61589891
rs148164126
rs10412462
rs76777800
rs28572049
rs77216559
rs29630
rs4360502
rs75066660
rs11766761
rs143131035
rs113580784
All
−0.15
−0.10
−0.05
0.00
0.05
0.10
MR leave−one−out sensitivity analysis for
'GCST90274828.csv' on 'outcome'
